# Electroacupuncture Alleviates Hyperalgesia by Regulating CB1 Receptor of Spinal Cord in Incisional Neck Pain Rats

**DOI:** 10.1155/2021/5880690

**Published:** 2021-12-18

**Authors:** Junying Wang, Jinling Zhang, Yonghui Gao, Yu Chen, Chenglin Duanmu, Junling Liu

**Affiliations:** Department of Physiology, Institute of Acupuncture and Moxibustion, China Academy of Chinese Medical Sciences, Beijing 100700, China

## Abstract

Acupuncture therapy is effective in relieving postoperative pain of neck surgery, but its underlying mechanisms remain largely unknown. This study, in the incisional neck pain rat model, was designed to explore whether the endocannabinoid receptor 1 (CB1) in the cervical spinal cord is involved in the analgesic effect of electroacupuncture (EA) or not.The incisional neck pain model was established by making a longitudinal incision and applied EA treatment of Futu (LI18), Hegu-Neiguan (LI4-PC6), or Zusanli-Yanglingquan (ST36-GB34) for pain relief. The results showed that EA LI18 and EA LI4-PC6 effectively relieve allodynia caused by neck incision, which was obviously better than EA ST34-GB34 (*P* < 0.05). After EA, the expression levels of CB1 mRNA at 4h in the EALI18 group, and 24 and 48h in both EALI18 and EALI4-PC6 groups, and those of CB1 protein at 4, 24, and 48h in the EALI18 group, and the immunoactivity of CB1 in both EALI18 and EALI4-PC6 groups at 4h were significantly upregulated in contrast to those of the model group (*P* < 0.05). EA of either acupoint group had no effect on the expression of CB2 protein (*P* > 0.05). Moreover, the antinociceptive effect of EA was reversed by AM251 (CB1 antagonist). Immunofluorescence dual staining showed that CB1 expressed in astrocytes in the superficial layer (laminae I and II) of dorsal horns of the cervical spinal cord. Therefore, the findings of this study revealed that upregulation of CB1 expression in the cervical spinal cord contributes to the analgesic effect of EA in incisional neck pain rats. The CB1 receptor expresses on astrocytes.

## 1. Introduction

Postsurgical pain is a common acute pain and frequently seen in many common operations, such as thyroid surgery, inguinal hernia repair, and breast and chest surgery. About 86% of the surgery patients suffer from moderate to severe postoperative pain [1], and more than 10–50% of them may experience persistent postoperative pain [2], seriously affecting their daily life quality. Therefore, more and more attention has been paid to effective postoperative management, but the existing anesthetics or analgesics have obvious limitations and adverse reactions, including tolerance, dependence, gastrointestinal dysfunction, cognitive impairment, narrow treatment window, and patients' low satisfaction with analgesics [3, 4]. As a result, more and more doctors and patients seek nonpharmaceutical treatment to relieve the pain. A large number of studies have shown that electroacupuncture (EA) can significantly relieve postsurgical pain, with fewer side effects [5–11], but its mechanism is still unclear.

The endocannabinoid system, consisting of two main ligands N-arachidonoylethanolamine (AEA) and 2-arachidonoylglycerol (2-AG), which are considered to be the main endogenous ligands of the two corresponding receptors: cannabinoid receptor 1 (CB1) and cannabinoid receptor 2 (CB2), is one of the key endogenous systems for processing pain sensation. The CB1 is the prominent subtype in the central nervous system (CNS), including spinal cord, thalamus, periaqueductal grey (PAG), amygdala, and rostroventromedial medulla [12, 13] and also exists in the dorsal root ganglion, at presynaptic sites throughout the peripheral system and CNS [14], whereas CB2 is found principally (but not exclusively) on immune cells. Both of them participate in nociceptive processing [14]. In the spinal dorsal horns (DHs), CB1 is found to express on microglia, oligodendrocytes, astrocytes, and interneurons [15, 16]. Intrathecal injection of CB1 antagonist caused obvious hyperalgesia in mice [17] and enhanced electric stimulation c-fiber induced discharge of neurons in the spinal dorsal horns (DHs) [18]. In incisional paw pain rats, the spinal anandamide level was evidently decreased at the early time, being consistent with the maximum mechanical hypersensitivity, and restored to the baseline level as the noxious behavior subdued. Administration of CB1 and CB2 antagonists blocked the reduction of postsurgical allodynia and caused persistent overexpression of glial fibrillary acidic protein (GFAP) and p-p38 in astrocytes [19]. Recent studies have shown that astroglial CB1 receptors control synaptic transmission and plasticity [20]. CB1 receptors in astrocytes may be aslo involved in the antihyperalgesic action of exogenous cannabinoids [21].

It has been demonstrated that EA has both analgesic and anti-inflammation effects via regulating the endocannabinoid system, including activation of CB1 and CB2, and also can reduce the adverse reactions caused by exogenous administration of cannabinoid [22]. The expression levels of CB1 and CB2 genes were upregulated 6 hours after EA treatment in temporomandibular arthritis rats [23]. Application of CB1 selective antagonist AM251 significantly reversed the analgesic effect of EA, and application of CB2 selective antagonist AM630 significantly reversed the anti-inflammatory effect of EA [23]. Activation of spinal CB1 and upregulation of spinal CB1-ERK1/2 signaling could enhance the antinociceptive effect of EA in morphine-induced hyperalgesia rats [24]. Although these outcomes have detailed the functions of CB1 and CB2 in mediating the analgesic effect of EA, their association with the effect of EA in inducing the reduction of incisional neck pain (INP) has not been reported. Our past studies displayed that EA Futu (LI18) could suppress neck incision-induced thermal hyperalgesia which may be related to its functions in upregulating the expression levels of GABA and its receptors [6] and downregulating P2X7R/fractalkine/CX3CR1 signaling [25] SP/NK1, COX-1, and PGE2 levels [26] in the cervicospinal DHs. Immunofluorescence double staining showed that GABA was expressed on astrocytes and neurons, and GABA-B was expressed only on neurons [6]. However, it was reported that the activated CB1 and GABA have a role in dorsal horn pain-controlling circuits, and spinal antinociceptive effects of GABA(B) receptor agonists are likely through endocannabinoid modulation [27, 28].

Therefore, this study was designed to select the appropriate acupoint with the best analgesic efficacy for INP, then, to observe the dynamic effect of EA on the expression of CB1 mRNA and protein in DHs of the cervical spinal cord, and at last, to verify the effect of CB1 in mediating EA-induced analgesic effect by intrathecal and intraperitoneal administration of CB1 antagonist.

## 2. Method

### 2.1. Ethical Statement

All experimental protocols and animals' treatment were approved by the Animal Welfare and Use Ethics Committee of the Institute of Acupuncture and Moxibustion, China Academy of Medical Sciences (Approval Number: 20140014); all experimental methods were conducted in accordance with the welfare ethics and “3R” principles of laboratory animals and conformed to the Guidelines for the Care and Use of Laboratory Animals made by the National Institutes of Health (NIH Publication No.85-23, revised in 1985).

### 2.2. Animals and Grouping

A total of 316 male Sprague-Dawley (SD) rats (200–220 g in weight), purchased from the Experimental Animal Center of China Academy of Medical Sciences (License No.: SCXK (Beijing) 2014-0013), were housed in the standard animal room with 22 ± 2°C and 12 h : 12 h of the light-dark cycle. The rats were given free access to food and water and acclimatized to the laboratory conditions for 7 days before the experiment.

These rats were randomly divided (by using a random digital table) into 5 groups, control, model, EA Futu (LI 18), EA Hegu (LI 4)-Neiguan (PC 6) group, and EA Zusanli (ST 36)-Yanglingquan (GB 34), which were further randomized into three subgroups: 4 (with 16 rats in each subgroup), 24(with 21 rats in each subgroup), and 48(with 16 rats in each subgroup). In order to verify the role of CB1 receptor in mediating EA analgesia, the rats were randomly divided into two groups, DMSO + EA and CB1 antagonist (AM251) + EA, which were further randomized into two subgroups: intraperitoneal injection(I.P.) and intrathecal injection (I.T.), with 12 rats in each subgroup. Three rats were excluded due to dyskinesia.

### 2.3. Establishment of Incisional Neck Pain Model

The day before the operation, the rat's neck hair was removed using appropriate amount of depilatory cream. Mild anesthesia was performed with isoflurane (0.5–1% oxygen) inhaled from the nasal cone using a desktop animal anesthesia ventilator system (VME, Matrix Company, USA). According to our previous study [26], the rat's baseline thermal pain threshold was detected first, and then, a 1.5 cm longitudinal incision was made along the midline of the neck, followed by repeated blunt dissection stimulation along the bilateral sternohyoideus around the thyroid gland regions for 30 min with a pair of forceps. The incision was then sutured in layers with a piece of 4.0 surgical catgut. After surgery, the rats were placed back to the cages for recovery. Rats in the sham operation (control) group were similarly anesthetized, but without incision and dissection stimulation. The neck incisional pain models were measured by the thermal pain threshold as our previous study [26].

### 2.4. Electroacupuncture Intervention

According to the graphical representation and word description of rat acupoints and human acupoints in book “*Experimental Acupuncture and Moxibustion*” [29], Futu (LI 18) is located on the lateral side of neck, between the sternal head and clavicular head of the sternocleidomastoideus, at the level of the 4th cervical vertebra, Hegu (LI 4) is located between the 1st and 2nd metacarpals of the forelimb; Neiguan (PC 6) is located on the ventral side of the forelimb, about 3 mm from the transverse crease axis of wrist, between the ulnar and radius; Zusanli (ST 36) is located on the lateral side of the knee joint of the hind limb, 5 mm below the head of fibula; and Yanglingquan (GB 34) is located 5 mm superior lateral to Zusanli (ST 36).

Under anesthesia with 1.5% isoflurane, the rats of the three EA groups received the insertion of acupuncture needles (No. 32, Suzhou Acupuncture Products Co., Ltd, China) into bilateral LI18, LI4, PC6, or bilateral ST36 and GB34, to a depth of about 2–4 mm. After needle insertion, the needle handles were connected to the output terminals of the HANS apparatus (HANS-200A, Jisheng Medical Technology, Co., Ltd., China) for stimulating the acupoints with a frequency of 2/100 Hz and intensity of 1 mA for 30 min during neck incision and 20 h and 44 h after incision.

### 2.5. Measurement of Thermal Pain Threshold

The rats in each group were wrapped in special cloth bags, with their necks exposed. The thermal pain threshold of the neck incision area was measured with Tail Flick (37360, UGO Basile, Italy) before operation, and 4 and 24 h after surgery. A radiant heat lamp was focused on the center of the neck incision area; when the rat rapidly removed its neck from the heat source, it was recorded as the thermal pain threshold (TPT, i.e., neck withdrawal latency, NWL). Each rat was tested 3 times with an interval of about 5–10 min, and the average value was taken. The researcher who analyzed the TPT results was unaware of the grouping and not involved in the acupuncture process either.

### 2.6. Intrathecal Tube Implantation

Intrathecal tube surgery was performed under inhalational isoflurane (1–2% oxygen) anesthesia. The lumbar vertebrae (L5-L6) were exposed. A polyethylene (PE) 10 catheter (OD 0.61 mm, ID 0.28 mm, Smiths Medical, UK), prefilled with 0.9% sterile saline, was inserted into the narrow space between L5 and L6. According to the improved catheterization without laminectomy described previously [30], the PE catheter was advanced cephalically about 7.5 cm into the subarachnoid space of cervical vertebrae C2-C5. The local muscle and skin were sutured in layers with a piece of surgical catgut. The outside end of the catheter, exposed for about 2-3 cm and closed with a cautery pen, was embedded into the muscle layer and fixed. The rats were allowed to have a recovery period of 5–7 days before the next experimental procedure. At the end of the experiment, the position of the catheter was verified by lidocaine injection. Only those rats with transient forelimb paresis after lidocaine injection were included. Five days after surgery, the Salzman scale [31] was used to detect the hind limb motor function in each group, and 3 rats with a score lower than 6 points were excluded due to motor function defect.

### 2.7. Drug Administration

One week after intrathecal tube implantation, the rats were anesthetized with isoflurane and received an intrathecal injection of 10 *µ*L CB1 antagonist AM251 solution (10 mg/mL, dissolved in DMSO) or 10 *µ*L DMSO (*n* = 12/group), once a day for 5 days before the incision operation. Other 24 rats (without i.t. catheter) received an intraperitoneal injection of 200 *μ*L CB1 antagonist AM251 at doses of 3 mg kg^−1^ of body weight (dissolved in DMSO) or 200 *μ*L DMSO (*n* = 12/group), once daily for 5 days. All rats that underwent neck incision and bilateral Futu (LI 18) were stimulated by EA. The thermal hyperalgesia in the neck incision area was measured before and 4, 24, 48, and 72 hours after the operation.

### 2.8. Western Blot

Rats in each group were anesthetized by the administration of pentobarbital sodium (35 mg/kg weight, i.p.). The tissue sample of the cervical spinal cord (C2-C5) was taken out on ice and placed into a 1.5 m cryogenic microtube to be rapidly frozen in liquid nitrogen after semitransection and stored at −80°C till use. The frozen tissue sample (100 mg) was placed in the RIPAlysis buffer with protease inhibitor cocktail (Roche, Mannheim, Germany), and the target protein was extracted through proteolysis by using a tissue homogenizer. The concentration of the protein was detected using the bicinchoninic acid (BCA) method with a bovine serum albumin standard. The same amount of protein samples were separated in 5%, 12%, and 15% sodium dodecyl sulfate polyacrylamide gel electrophoresis (SDS-PAGE) and transferred to a polyvinylidene difluoride (PVDF) membrane (0.2 or 0.45 *μ*m, Millipore Corporation, Billerica, MA, USA). The membrane was blocked in 5% bovine serum albumin tris-buffered saline plus Twine (BSA-TBST) at room temperature (RT) for 1 h and then incubated with primary antibody: rabbit anti-CB1 (1 : 2000, ab137410, Abcam, UK), rabbit anti-CB2 (1 : 2000, ab3561, Abcam, UK), and mouse anti-*β*-actin (1 : 5000, YM3028, Immunoway, USA) at 4°C overnight. After washing with TBST 3 times, the membrane was incubated with horseradish peroxidase-conjugated goat anti-rabbit antibody (1 : 500, ab6721, Abcam, UK) and goat anti-mouse IgG (1 : 10,000, Jackson immunoresearch Laboratories, West Grove, PA, USA) at room temperature for 2 h. Standard X-ray film (Eastern Kodak Co., Rochester, NY, USA) was then used to show the immunoreactive bands through the enhanced chemiluminescence (Amersham Biosciences UK Limited, Buckinghamshire, England). The level of band intensity was quantified by using Image-Pro Plus software (Media Cybernetics, Silver Spring, MD). Band densities were normalized to individual *β*-actin internal controls.

### 2.9. Quantitative Real-Time PCR

Total RNA was extracted from the sample tissue of spinal dorsal portion (C2-C5) by using Trizol (Invitrogen, Carlsbad, CA). The reverse transcription of cDNA was obtained using the PrimeScript-TM Reagent Kit (Takara Bio, Shiga, Japan). The primers were designed by using primer 3.0 and synthesized by Sangon (Shanghai, China). The primer sequences are shown in Table 1. A fluorescence quantitative PCR reaction system was configured, and the tissue sample was added to each well of the 96-well plate and then placed onto the real-time quantitative fluorescence PCR system (ABI7500, Applied Biosystems, USA), to construct the real-time PCR reaction system by using cDNA samples. PCR was performed under the following conditions: 95°C, 30 seconds; 40 PCR cycles (95°C, 5 seconds; 60°C, 40 seconds (to collect fluorescence), followed by 95°C, 10 seconds; 60°C, 60 seconds; 95°C, 15 seconds to establish the fusion curve of PCR products. The samples with target gene and reference gene were run on the same plate to avoid between-run variations, and each sample was tested for three complex holes. The relative expression of genes was calculated in accordance with the 2^−∆∆CT^ method.

### 2.10. Immunofluorescence Multiple Labeling

Under deep anesthesia with pentobarbital sodium (35 mg/kg weight, i.p.), the rats received a transcardiac perfusion with normal saline (250 mL) and then with 4% paraformaldehyde solution (200 mL) containing 1.5% picric acid in 0.1 M PB (pH 7.4). The cervical spinal cord segment (C2-C5) was removed and postfixed in the same fixative solution overnight and then dehydrated with 30% sucrose solution overnight. The spinal cord sample tissue was cut into sections (40 *μ*m) by using a cryostat microtome (Thermo, Microm International GmbH, Germany). Free-floating tissue slices were cultured in primary antibody, mouse anti-GFAP (1 : 1000, 3670s, Cell Signaling Technology, USA) and rabbit anti-CB1 (1 : 1000, ab23703, Abcam, UK) at 4°C overnight and then cultured in secondary antibodies, donkey anti-mouse IgG antibody conjugated Alexa Fluor 488 (1 : 500, A21202, Life Technologies, USA) and donkey anti-rabbit IgG antibody conjugated Alexa Fluor 594 (1 : 500, A32754, Life Technologies, USA) at room temperature for 2 h. Finally, the slices were mounted onto glass slides and sealed with cover glasses. Three slices (within the dorsal horn) of each rat were photographed by using a multifunctional confocal microscope (Olympus, Tokyo, Japan). Five regions in the same part of the spinal DHs were randomly selected from a slice at the same magnification, and the immunofluorescence intensity of CB1 was measured by using NIS Elements software (Nikon, Tokyo, Japan). The mean intensity of five regions in each tissue slice (3 slices per rat) was calculated to be used as the fluorescence intensity value.

### 2.11. Statistical Analysis

All data were expressed as mean ± standard deviation (mean ± SD) and analyzed with SPSS20.0 statistical software. All data were subjected into the homogeneity test of variances first. Data of TPT were analyzed by two-way repeated measures analysis of variance (ANOVA), and data of quantitative real-time PCR and WB and immunofluorescence intensity were analyzed by one-way ANOVA, followed by LSD tests for comparison between two groups, or Student's *t*-tests. Statistical significance was defined as *P* < 0.05.

## 3. Result

### 3.1. Electroacupuncture Alleviates the Neck Incisional Pain

**Figure 1:**
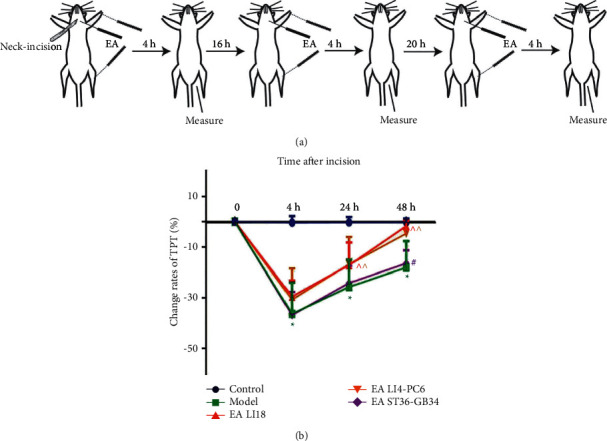
EA increases thermal pain threshold (TPT) in rats with neck incisional pain. (a) Schematic diagram of experimental procedures of EA intervention and TPT measurements: TPT was measured before neck incision, and 4, 24, and 48 h after EA intervention. (b) Histograms showing the effect of EA at Futu(LI18), Hegu (LI14)-Neiguan(PC6), and Zusanli(ST36)-Yanglingquan(GB34) on the change rate of TPT at different time points after modeling (mean ± SD, *n* = 13/group). The change rate of TPT (TPT%) = (TPT after neck incision − TPT before neck incision)/TPT before neck incision × 100%. Repeated measures ANOVA revealed that TPT% was significantly decreased from 4 to 48 h after modeling (vs the control group) and considerably increased at 24 and 48 h after EA of LI 18 and LI4-PC6, rather than EA of ST36-GB34 (vs the model group). ^*∗*^*P* < 0.05, vs the control group, ^^^*P* < 0.05, vs the model group, ^#^*P* < 0.05, vs the EA LI18 group, all by the LSD test.

In order to observe the effect of EA on incisional neck pain, the TPT was measured before the neck incision and at 4, 24, and 48 h after the incision. The change rates of TPT = (TPT after neck incision − TPT before neck incision)/TPT before neck incision × 100%. Compared with the normal control group, the change rates of TPT in the model group were significantly reduced at 4, 24, and 48h (*P* < 0.05, Figure 1(b)), indicating an occurrence of hyperalgesia in the local incisional region. Compared with the level of 4h after neck incision, the change rates of TPT were increased gradually at 24 and 48 h. Compared with the model group, the change rates of TPT in the EA LI18 and EA LI4-PC6 groups were significantly increased at 24 and 48 h after incision (*P* < 0.05, Figure 1(b)), indicating that EA at LI18 and LI4-PC6 could effectively relieve the incisional neck pain. There was no significant difference between the model and EA ST36-GB34 groups in the TPT levels at the 3 time points (*P* > 0.05).

### 3.2. EA of LI18 Upregulates Expression of CB1 Protein

**Figure 2:**
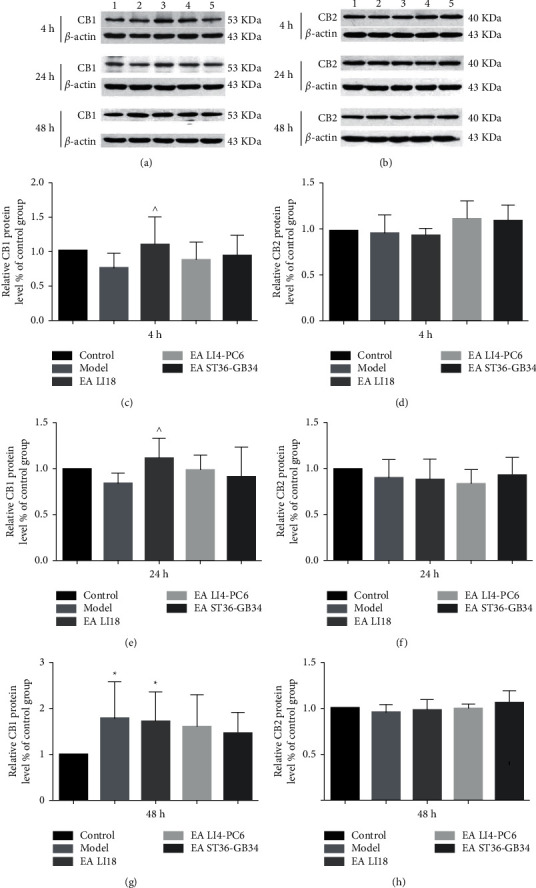
Effect of EA on the expression levels of endocannabinoid receptor CB1 (a, c, e, g) and CB2 (b, d, f, h) proteins at the 3 time points after modeling in the dorsal portion of the cervical spinal cord (C2-C5) in neck incisional pain rats. Representative gel images showing the protein level of CB1 (a) and CB2 (b) receptors in the cervical spinal cord tissues in different groups: (1) control group, (2) model group, (3) EA LI18 group, (4) EA LI4-PC6 group, and (5) EA ST36-GB34 group. The expression of CB1 protein was significantly increased at 48 h in the model group (vs the control group), and at 4, 24, and 48 h in the EA LI18 group (VS the model group), and the expression levels of CB2 protein at 4, 24, and 48 h had no notable changes after modeling (vs the control group) and after EA of the 3 acupoint groups (vs the model group). Data are expressed as means ± SD (*n* = 8). ^*∗*^*P* < 0.05, vs the control group, ^^^*P* < 0.05, vs the model group.

The protein expression levels of CB1 and CB2 in the spinal dorsal part at 4, 24, and 48 h after neck incision and after EA were observed, respectively. In comparison with the normal control group, the expression of CB1 protein only at 48 h after neck incision was significantly increased in the model group (*P* < 0.05, Figure 2(g)). Compared with the model group, the expression levels of CB1 protein at 4 and 24 h after neck incision were significantly increased in the EA LI18 group (*P* < 0.05, Figures 2(c) and 2(e)). No significant changes were found in the expression levels of CB1 at 4, 24, and 48 h in both the LI4-PC6 and EA GB36-GB34 groups in contrast to the model group (*P* > 0.05, Figures 2(c), 2(e), 2(g)), and such is the case in the expression levels of CB2 at the 3 time points after neck incision in both model and 3 EA groups (*P* > 0.05, Figures 2(d), 2(f), 2(h)).

**Figure 3:**
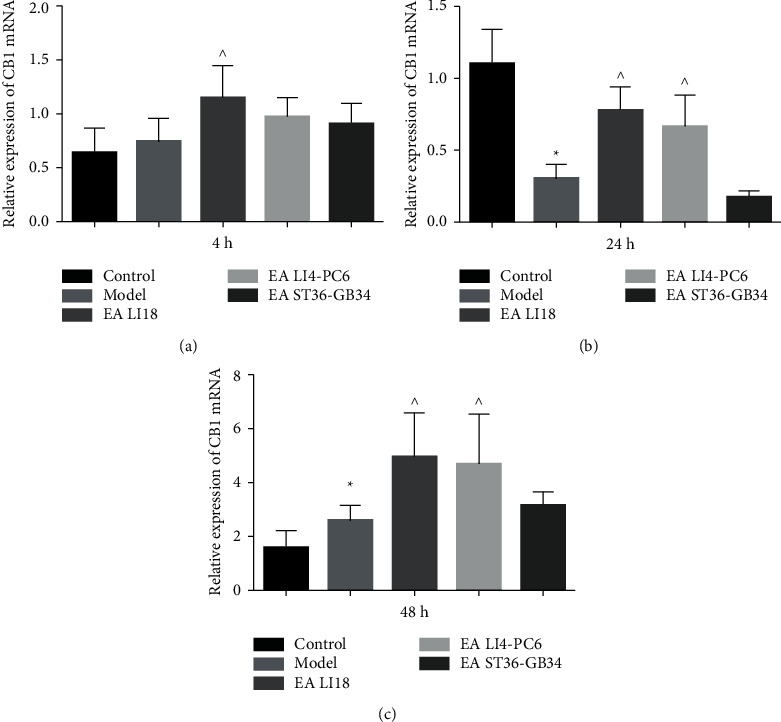
Effect of EA on the expression of endocannabinoid receptor CB1 mRNA at 4 (a), 24 (b), and 48 h (c) after modeling in the dorsal portion of the cervical spinal cord in neck incisional pain rats. The expression levels of CB1 mRNA were evidently decreased at 24 h and remarkably increased at 48 h after modeling (vs the control group), and considerably upregulated at 4h in the EALI18 group, at 24 and 48 h in both EALI18 and EALI4-PC6 groups, rather than in the EAST36-GB34 group (vs the model group). Data are expressed as means ± SD (*n* = 8). ^*∗*^*P* < 0.05, vs the control group, ^^^*P* < 0.05, vs the model group.

Regarding the outcomes of QRT-PCR, the mRNA levels of CB1 in the cervical spinal dorsal portion were significantly downregulated at 24h after neck incision (*P* < 0.05, Figure 3(b)) but considerably upregulated at 48h in the model group in contrast to the control group (*P* < 0.05, Figure 3(c)). Compared with the model group, the CB1 mRNA expression levels at 4 h in the LI18 group and at 24 and 48 h in both EA LI18 and EALI4-PC6 groups were significantly increased (*P* < 0.05, Figures 3(a)–3(c)), whereas no significant changes were found at 4h in the EA LI4-PC6 group and at the three time points in the EA ST36-GB34 group (*P* > 0.05).

### 3.3. EA Upregulates Immunoactivity of CB1 Expressing in Astrocytes

**Figure 4:**
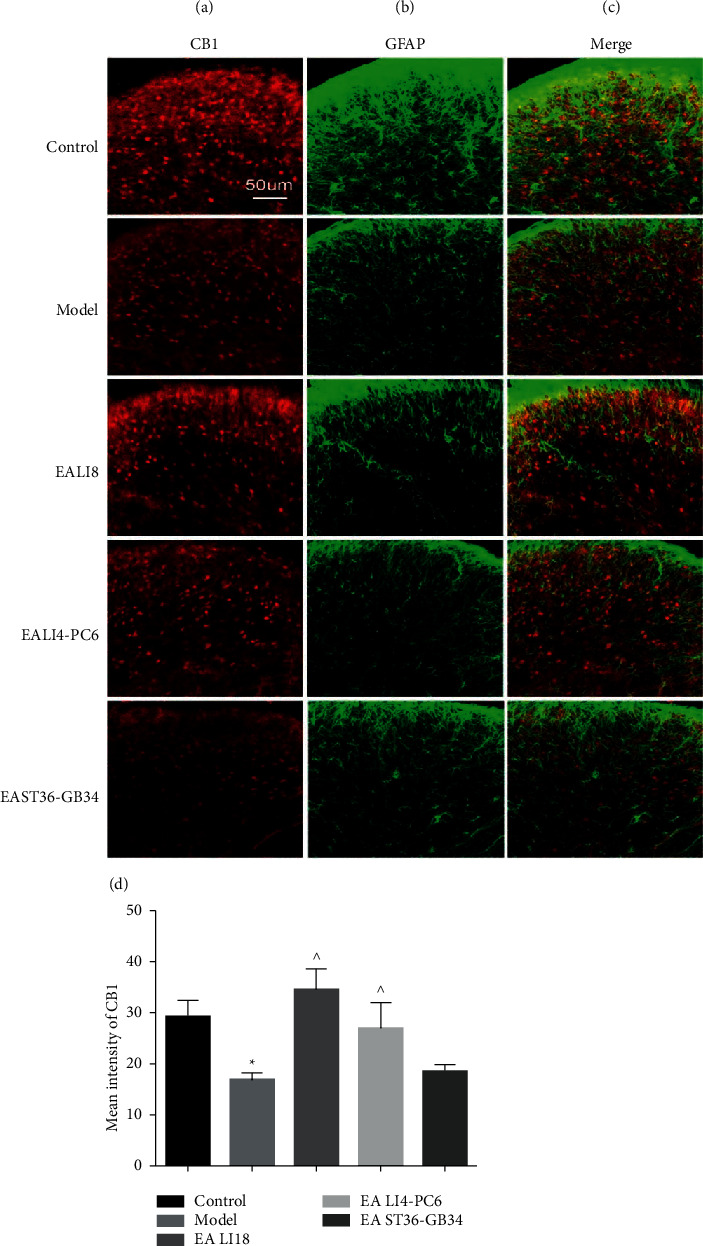
Effect of EA on the immunoactivity of endocannabinoid receptor CB1 at 24 h after modeling in neck incisional pain rats. Double immunolabeling of CB1 receptor and glial fibrillary acidic protein (GFAP, marker of astrocytes) in the superficial layer (laminae I and II) of cervical spinal dorsal horns (DHs): (a). CB1 positive labeling (red), scale bar, 50 *μ*m; (b) GFAP-positive labeling (green), (c) double-labeled cells(yellow). (d): Histograms showing the intensity of the immunoactivity of CB1 receptors in the 5 groups. One-way ANOVA revealed that the expression level of CB1 was notably downregulated in the model group (vs the control group) and significantly upregulated in both EALI18 and EALI4-PC6 groups but not in the EAST36-GB34 group (vs the model group). Data are expressed as means ± SD (*n* = 5). ^*∗*^*P* < 0.05, vs the control group, ^^^*P* < 0.05, vs the model group.

Compared with the normal control group, the mean immunofluorescence intensity (immunoactivity) of cannabinoid receptor CB1 at 24 h after neck incision in the superficial layer of cervical spinal DHs was significantly decreased in the model group (*P* < 0.05, Figure 4(d)). Compared with the model group, the levels of immunoactivity of CB1 in the spinal DHs were significantly increased in both EA LI18 and EA LI 4-PC6 (*P* < 0.05, Figure 4(d)), but rather than in the EA ST36-GB34 group (*P* > 0.05).

In order to dissect the roles of the CB1 receptor on astrocytes during EA, the colocalization and immunoreactivity of CB1 and GFAP in each group were investigated. In the control group, CB1 was expressed on astrocytes in the spinal DHs, and after neck incision surgery, nearly no co-expression of CB1 and GFAP was visualized in each slice of the observed spinal cord. In the EA LI18 group, the co-expression of CB1 and GFAP was found in laminae I and II of the cervical spinal DHs, whereas no co-expression was visualized in both EA LI4-PC6 and EA ST36-GB34 groups. These results suggest that EA at LI18 upregulated the expression of CB1 in astrocytes, which may contribute to the process of EA analgesia.

### 3.4. CB1 Receptor Antagonist Weakens the Analgesic Effect of EA

**Figure 5:**
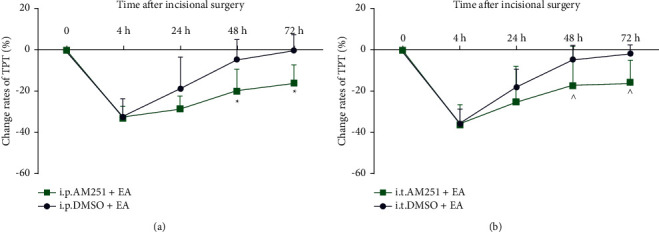
Intraperitoneal (a) and intrathecal (b) injection of CB1 antagonist (AM251) decreased the analgesic effect of EA LI18. Repeated measures ANOVA revealed that the PTP was significantly decreased after modeling (vs preneck incision) and obviously increased thereafter (vs 4 h), with the change rate being notably higher at 48 and 72 h in the DMSO + EA group than in the AM251+EA group. Data are expressed as means ± SD (*n* = 12). ^*∗*^*P* < 0.05, vs the i.p.DMSO + EA group, ^^^*P* < 0.05, vs the i.t. DMSO + EA group.

In order to confirm the involvement of CB1 receptors in the analgesic effect of EA, both the intraperitoneal injection and intrathecal injection of CB1 antagonist (AM251) were conducted in the present study. The results showed that either intraperitoneal or intrathecal injection of DMSO did not influence the increase in the change rates of TPT after EA at LI18. It indicated that administration of DMSO had no impact on the analgesic effect of EA. Compared with the DMSO + EA group, the change rate of TPT had no marked alteration at 4 and 24 h after neck incision (*P* > 0.05) and significantly decreased at 48 h and 72 h after intraperitoneal or intrathecal injection of AM251 (*P* < 0.05, Figures 5(a) and 5(b)). These results indicated that the CB1 receptor antagonist AM251 but not DMSO significantly reversed the analgesic effect of EA in incisional neck pain rats.

## 4. Discussion

Postoperative pain caused by surgical injury is a major challenge for healthcare providers. Opioid analgesics are commonly used to treat postoperative pain. However, these drugs have some adverse side effects. Therefore, more and more attention has been paid to the management and treatment of postoperative pain. It was reported that percutaneous peripheral nerve stimulation can be used to treat acute postoperative pain of various types of surgeries [32]. Acupuncture intervention and transcutaneous electrical nerve stimulation (TENS), being similar to percutaneous peripheral nerve stimulation, have been proved to be effective for relieving postoperative pain of neck surgery [9, 10, 33], tonsillectomy [7, 11, 34], back surgery [5], dental procedures [7, 35], and knee replacement [7]. Our previous clinical study [33] also showed that EA of LI18 and LI4-PC6 had a good effect in inducing analgesia and controlling the mean arterial pressure (MAP) and heart rate (HR), and reduced the anesthetic dose required by patients undergoing thyroid surgery. In this study, the PTP of rats was significantly reduced after neck incision plus repeated topical mechanical separation stimulation and considerably increased at 24 and 48 h after EA of LI18 and LI4-PC6 rather than EA of ST36-GB34, which is consistent with the results of our previous studies [6, 25, 26, 36].

The spinal cord, the primary center of nociceptive signal processing, plays a critical role in the integration and modulation of nociceptive inputs before sending to the higher central neuronal networks. Endocannabinoid is well known to be an important neuroregulatory mediator for relieving different types of pain, including chemical, mechanical, thermal, neuropathic, inflammatory, and cancer pain [37–39]. Our results of the present study showed that after neck incision, the expression levels of CB1 mRNA and protein in the cervical spinal cord had no significant changes at 4h, but were significantly increased at 48h, and those of CB1 mRNA and immunoactivity were obviously decreased at 24 h. These inconsistent results about changes of the expression levels of CB1 mRNA and protein have no suitable reasons to explain, whereas after EA interventions, the expression levels of CB1 mRNA at 4 h in the EALI18 group, 24 and 48 h in both EALI18 and EALI4-PC6 groups, and those of CB1 protein at 4, 24, and 48 h in the EALI18 group were significantly upregulated, which may contribute to the analgesic effect of EA of LI18 and LI4-PC6 in neck incision pain rats. Verification experiments demonstrated that intrathecal and intraperitoneal injection of CB1 antagonist AM251 significantly inhibited the analgesic effect of EA LI 18 in incisional neck pain rats. These findings reveal the involvement of the CA1 endocannabinoid receptor of cervical spinal DHs in the analgesic effect of EA in neck incision pain rats for the first time and are similar to those of other research studies. For example, Zheng and his colleagues [24] reported that in intrathecal (IT) morphine-induced hyperalgesia (MIH) rats, EA of ST36-GB34 reversed the reduction of mechanical and thermal TPT, and IT injection of CB1 agonist (WIN55, 212-2) combined with EA induced a significant increase of mechanical and thermal TPT and a significant increase of CB1 protein level, whereas IT CB1 antagonist (SR141716) induced the opposite results. In knee osteoarthritis (KOA) mice, EA reversed modeling-induced reduction of CB1 receptor expression and endocannabinoid 2-AG level in the midbrain, and microinjection of CB1 receptor antagonist into vlPAG (projecting to rostral ventromedial medulla-spinal neurons) could reverse the effect of EA on pain hypersensitivity and diffuse noxious inhibitory control (DNIC) function [22]. Additionally, in experimental temporomandibular joint arthritis rats, EA upregulated the expression of CB1 and CB2 mRNAs; the administration of CB1 antagonist AM251 in the temporomandibular joint significantly reversed the antinociceptive effect of EA, and the injection of CB2 antagonist AM630 reversed the anti-inflammatory effect of EA [23]. In facial thermal pain rats, EA stimulation produced an antinociceptive reaction, which was antagonized by the pre-administration of AM 251, but not by AM 630, and pretreatment with an endocannabinoid metabolizing enzyme inhibitor (MAFP) and an anandamide reuptake inhibitor (VDM11) prolonged and enhanced the antinociceptive effect of EA [40]. Therefore, CB1 receptor plays an important role in mediating the antinociceptive effect of EA both in the central nervous system (in particular) and the peripheral tissues. Nevertheless, accumulated evidence [41, 42] supports that in the peripheral tissues, CB2 not CB1 induces an antinociceptive effect, possibly due to its widespread distribution in keratinocytes, macrophages, and T-lymphocytes in the epidermis and dermis of the inflamed skin tissue.

In the present study, the results of western blotting displayed that the expression of CB2 receptor protein had no significant changes at 4, 24, and 48 h after neck incision and after EA of the three acupoint groups, suggesting that CB2 in DHs of the cervical spinal cord may do not contribute to the EA's analgesic effect. Moreover, outcomes of immunofluorescence dual labeling showed that the co-expression of CB1 and GFAP was seen in the superficial layer (laminae I and II) of the spinal DHs in the control group and increased in the EA LI18 group, but nearly not visualized in the model, EALI4-PC6 and EAST36-GB34 groups, suggesting that the upregulated expression of CB1 in astrocytes in the cervical spinal DHs is involved in the process of EA analgesia in incisional neck pain rats. The distribution of CB1 receptor in the spinal DHs is consistent to Svízenská's and colleagues' results that CB1 receptors are densely expressed on the superficial layers of spinal dorsal horns, the dorsal root ganglia, and the peripheral terminals of primary afferent neurons, and within the pain descending pathway [43] and Alkaitis's and colleagues' findings that Dual blockade of CB1 and CB(2) receptor signaling prevented the resolution of postoperative allodynia and resulted in persistent overexpression of spinal GFAP and phospho-p38 in astrocytes [19].

Regarding the specific effects of EA of the three acupoint groups, the results indicated that EA LI18 and EA LI4-PC6 are basically similar in upregulating the thermal TPT and the expression of CB1 mRNA at 24 and 48 h, and increasing the immunoactivity at 24h. EA of LI18 (not LI4-PC6) also significantly upregulated the expression levels of CB1 protein at 4, 24, and 48 h, and CB1 mRNA at 4 h. Hence, EALI18 is the best in the therapeutic effect, whereas EA of ST36-GB34 had no apparent impact on the pain behavior reaction and expression of CB1 and CB2 proteins and CB1 mRNA at the three time points. These results are identical to those of our past studies in the same neck incision pain rat model [6, 25, 26, 36]. The reason is that LI18, being close to the injured neck region, is located at the same nerve segment to the incisional pain source, whereas LI4 and PC6 are at the adjacent nerve segment to the incisional pain source. Therefore, the nearer the distance, the better is the therapeutic effect.

There are some limitations in this study. First, we only explored the distribution of the CB1 receptor in astrocytes and did not observe its distribution in neurons and microglia cells in the DHs of the cervical spinal cord, and their involvement in EA analgesia in the neck incision pain model is unclear. Second, the mechanisms of EA underlying relief of postoperative pain are very complicated and need research further in depth by using more and new indexes.

## 5. Conclusion

The present study for the first time demonstrated that the EA stimulation of LI18-induced upregulation of CB1 receptor expression in the dorsal portion of the cervical spinal cord may contribute to its antinociceptive effect in incisional neck pain rats. EA of LI4-PC6 can also effectively relieve postoperative pain in the same animal model. The therapeutic effect of EA at LI18 is relatively better in ameliorating pain behavior and upregulating expression of CB1 protein and gene, followed by EALI4-PC6 and EAST36-GB34 being the poorest. The CB1 receptor is found in astrocytes of the superficial layers of DHs of the cervical spinal cord. These findings may provide experimental evidence for relieving the postoperative pain of thyroidectomy surgery by acupuncture treatment.

## Figures and Tables

**Table 1:** The primer sequences.

The primer	Sequences	Length (bp)
CB1	Forward, 5′-TCCACCGTGAACCCCATCATCTA-3′ reverse, 5′-GCTGTGTTGTTGGCGTGCTTGT-3′	194
GAPDH	Forward, 5′-TTCCTACCCCCAATGTATCCG-3′ reverse, 5′-CCACCCTGTTGCTGTAGCCATA-3′	270

## Data Availability

The data used to support the findings of this study are included within the article.
